# Influence of Cerebral Vasodilation on Blood Reelin Levels in Growth Restricted Fetuses

**DOI:** 10.3390/diagnostics11061036

**Published:** 2021-06-04

**Authors:** Jara Pascual-Mancho, Pilar Pintado-Recarte, Carlos Romero-Román, Jorge C. Morales-Camino, Concepción Hernández-Martin, Julia Bujan, Miguel A. Ortega, Juan De León-Luis

**Affiliations:** 1 Department of Public and Maternal and Child Health, School of Medicine, Complutense University of Madrid, 28040 Madrid, Spain; jpascualm@salud.aragon.es (J.P.-M.); ppintadorec@yahoo.es (P.P.-R.); concepcion.hernadez@salud.madrid.org (C.H.-M.); jaleon@ucm.es (J.D.L.-L.); 2 Department of Obstetrics and Gynecology, University Hospital Gregorio Marañón, 28009 Madrid, Spain; 3 Health Research Institute Gregorio Marañón, 28009 Madrid, Spain; 4 Department of Obstetrics, Prenatal Diagnosis, Miguel Servet University Hospital, 50009 Zaragoza, Spain; 5 Laboratory of Clinical Biochemistry, Albacete Hospital, 02006 Albacete, Spain; crroman@sescam.jccm.es (C.R.-R.); jorgcmorales@hotmail.com (J.C.M.-C.); 6 Ramón y Cajal Institute of Healthcare Research (IRYCIS), 28034 Madrid, Spain; mjulia.bujan@uah.es; 7 Department of Medicine and Medical Specialties, Faculty of Medicine and Health Sciences, University of Alcalá, Alcalá de Henares, 28801 Madrid, Spain; 8 Pathological Anatomy Service, Central University Hospital of Defense-UAH, 28047 Madrid, Spain

**Keywords:** intrauterine growth restriction, perinatal neurodevelopment, reelin, hypoxia

## Abstract

Fetal growth restriction (FGR) is one of the most important obstetric pathologies. It is frequently caused by placental insufficiency. Previous studies have shown a relationship between FGR and impaired new-born neurodevelopment, although the molecular mechanisms involved in this association have not yet been completely clarified. Reelin is an extracellular matrix glycoprotein involved in development of neocortex, hippocampus, cerebellum and spinal cord. Reelin has been demonstrated to play a key role in regulating perinatal neurodevelopment and to contribute to the emergence and development of various psychiatric pathologies, and its levels are highly influenced by pathological conditions of hypoxia. The purpose of this article is to study whether reelin levels in new-borns vary as a function of severity of fetal growth restriction by gestational age and sex. We sub-grouped fetuses in: normal weight group (Group 1, *n* = 17), FGR group with normal umbilical artery Doppler and cerebral redistribution at middle cerebral artery Doppler (Group 2, *n* = 9), and FGR with abnormal umbilical artery Doppler (Group 3, *n* = 8). Our results show a significant association of elevated Reelin levels in FGR fetuses with cerebral blood redistribution compared to the normal weight group and the FGR with abnormal umbilical artery group. Future research should focus on further expanding the knowledge of the relationship of reelin and its regulated products with neurodevelopment impairment in new-borns with FGR and should include larger and more homogeneous samples and the combined use of different in vivo techniques in neonates with impaired growth during their different adaptive phases.

## 1. Introduction

Intrauterine growth restriction is among the most common obstetric syndromes; it can affect 3 to 10% of pregnancies when it is caused by placental insufficiency [1] The relationship between fetal growth restriction (FGR) and neurodevelopmental impairment is reflected by numerous epidemiological studies showing that affected new-borns (NBs) may present motor and/or cognitive disorders [2,3,4], following the trend reported by Baker in the last century with his theory of developmental origins of health and adult disease [5]. The mechanism by which neurodevelopmental disorders occur is unknown. Nutrient and oxygen deficiency due to the chronic decrease in umbilical flow at a moment of vital importance for neuronal proliferation and migration plays an important role in nervous system development [6]. The relationship between congenital heart disease, which chronically compromises prenatal brain flow, and the development of microcephaly and neurodevelopmental impairment has the same basis [7]. When noxa occurs during the migration phase, different phenotypes result in rodent models [8] and in neonatal models with a prenatal diagnosis of ischaemia by ultrasound [9]. The recent development of diffusion tensor magnetic resonance imaging has allowed for the assessment of white matter and connectivity in fetuses with FGR [10]. Doppler ultrasound and proton MRI spectroscopy have revealed that NBs with FGR present adaptive brain reorganization, with increased deterioration towards the basal ganglia [11], and altered brain metabolism, with reduced ratios of N-acetyl-aspartate:choline and N-acetyl-aspartate:creatinine [12,13].

The evaluation of metabolites in cord blood, particularly that of neurotrophins, is a distinct field of study [14]. Animal models have been important for studying the mechanism of involvement, allowing the direct study of the neurons in different brain regions, especially in the cerebral cortex, cerebellum, and hippocampus [8]. An important factor in perinatal neurodevelopment and one that is susceptible to alteration under hypoxic conditions is reelin [15], a glycoprotein of the extracellular matrix that is involved in the development of the laminated structures of the brain, mainly in the neocortex, spinal cord, hippocampus, and cerebellum [16]. During the prenatal stage, it is produced by the Cajal-Retzius neurons of the marginal zone, the hippocampus, and the external granular layer of the cerebellum [17]. It is responsible for radial neuronal migration and cortical lamination, although its role in growth and axon guidance has also been studied since it has been detected in the interneurons of white matter [18]. Mutations in the reelin gene produce the reeler phenotype in mice, which is characterized by a defect in brain lamination, and autosomal recessive lissencephaly in humans. In mouse embryo studies, hypoxic injury more intensely modified the expression of genes involved in synaptic function, neuronal transmission, and GABAergic interneuron migration, such as the reelin gene [19,20], which can be a candidate link between prenatal hypoxia and the risk for neurologic and mental health conditions such as schizophrenia and autism. A signaling pathway such as this one, which is involved in both development and brain plasticity, is an excellent target for the study of neurodevelopmental disorders, hence the increasing number of studies addressing the implications of intrauterine partial nutrient deficiencies, inflammation, or maternal infection, which modify the expression of the reelin gene [21,22] and may be the basis for schizophrenia, autism, or bipolar disorder, among other diseases [23].

Our objective is to analyze whether there is an association of reelin serum levels in cord blood of NBs with fetal growth restriction and its different growth restriction stages.

## 2. Patients and Methods

### 2.1. Selection of Patients and Evaluation of Fetal Growth

The project involved pregnant women cared for at the Maternidad del Hospital Universitario Gregorio Marañón (Maternity Ward of Gregorio Marañón University Hospital). The project was approved by the Clinical Research Ethics Committee (89/09). Gestational age was based on the first-trimester ultrasound using the craniocaudal length. Cases were selected after the estimated fetal weight (EFW) was determined by ultrasound using Hadlock equation 4, and the EFW percentile was classified according to our own reference population tables. When the EFW was below the 10th percentile, in accordance with the ISUOG FGR criteria [24], the Doppler ultrasound was performed to assess the pulsatility index of the umbilical artery, middle cerebral artery, and uterine artery, measured abdominally according to the Ciobanu [25], Gomez [26] reference charts, respectively. Doppler was not assessed on fetuses with adequate gestational age weight. Birth weight was obtained in the first 12 h of birth. Cases with congenital anomalies, clinical chorioamnionitis, illicit drug or alcohol use during pregnancy, or poor gestational control were excluded. The study was approved by the hospital’s ethics committee.

The subjects were divided into three study groups: Group 1: Appropriate for gestational age (AGA), EFW => 10 percentile; Group 2: FGR fetuses, EFW < 10 with a normal umbilical artery (UA) and pathological middle cerebral artery (MCA) (> p5); and Group 3: FGR fetuses (EFW < 10) with pathological UA (> p95) independent of the state of the MCA.

### 2.2. Sample Collection, Initial Processing, and Storage

The samples were collected from the umbilical vein after the delivery of the NB, just prior to delivery of the placenta, and were deposited in standard clinical lithium heparin tubes. The blood was centrifuged within the first hour of birth at the Biochemistry Department and stored in a Thermo Scientific Fisher Forma freezer at −86 °C. Thawing was performed at room temperature and occurred only once. The storage recommendations of Lugli et al. [27] were followed until gels formed, and antibody disposition was performed within the 5-year maximum.

### 2.3. Determination of Reelin in the Umbilical Vein through Western Blotting

Sample preparation: One milliliter of each test plasma sample was diluted in 30 mL of loading buffer (Laemmli buffer composed of 2 M Tris pH 6.8, 6 M urea, 15% b-mercaptoethanol, 0.003% bromophenol blue, and 3% SDS). The samples were heat-denatured for 3 min at 95 °C.

Protein separation was performed by electrophoresis on 6% SDS-polyacrylamide (Bio-Rad, Hercules, CA, USA) gels in the Mini Protean system (Bio-Rad) for 130 min at 120 volts, followed by wet transfer to a PVDF membrane (Millipore, Amsterdam, The Netherlands) and the use of the Mini Trans-Blot Cell system (Bio-Rad) for 15 h at 15 volts at 4 °C. Membrane blocking was carried out with 0.1% PBS Tween 20 (PBSt) with 5% milk powder for 1 h at room temperature, after which the samples were washed with PBSt before overnight incubation at 4 °C with the anti-reelin antibody a.a. 164–189 mreelin, clone 142 (Calbiochem, Millipore, Amsterdam, The Netherlands), diluted in PBSt with 1% milk. After incubation, three 5-min washes with PBSt were performed, and the samples were finally incubated for one hour at room temperature with horseradish peroxidase (HRP) goat anti-mouse (IgG) secondary (Santa Cruz Biotechnology, Santa Cruz, CA, USA) prepared in blocking solution. After three additional 5-min washes with PBSt, the membrane was revealed by chemiluminescence using enhanced chemiluminescent (ECL) reagent (Bio-Rad). Equal protein loading was estimated by re-probing the membranes with mouse anti-ceruloplasmin E-9 (Santa Cruz Biotechnology, Santa Cruz, CA, USA). No difference was observed between the ceruloplasmin content of the samples, so it was used only as a loading control, not for signal normalization, which refers to the initial sample volume. Signal detection was performed using the Image Reader LAS-4000 system (Fujifilm, Valhalla, NY, USA), and densitometric analysis was performed using ImageJ software. Briefly, the image is transformed into an 8-bit grayscale image; the same area containing the bands in each sample is selected. ImageJ draws a profile plot of each sample area selected, where peaks correspond with dark lines (protein band). Each peak is then labeled with its size and expressed as a percentage of the total size, and it is now possible to compare relative gray-density areas between different control groups and affected individuals; therefore, the data obtained do not include international units. Data were obtained for several molecular weights, 160 kDa, 310 kDa, and 420 kDa, and for the total sample. These data were analyzed in total, according to the methods described in the literature. [27,28].

**Figure 1:**
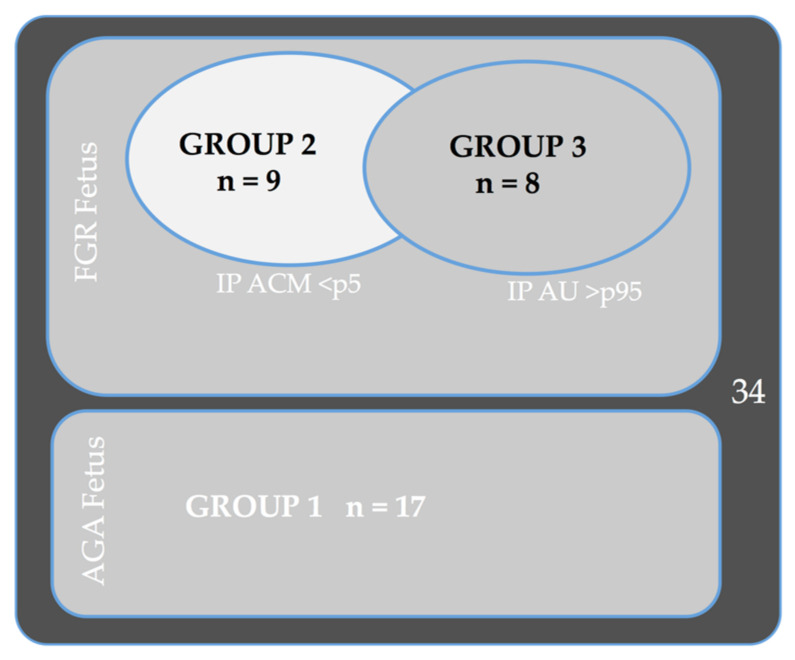
Descriptive groups.

Regarding the relationship between reelin concentration and FGR, three groups of fetuses were created. Group 1: Appropriate for gestational age (AGA), EFW => 10 percentile; Group 2: FGR fetuses, EFW < 10 with normal umbilical artery (UA) and pathological middle cerebral artery (MCA) (> p5); and Group 3: FGR fetuses (EFW < 10) with pathological UA (> p95) independent of the state of the MCA. (Figure 1)

For the statistical analysis, IBM SPSS Statistics V21.0 (Armonk, NY, USA) was used. The sample’s distribution was evaluated with the Shapiro–Wilk test and indicated a normal distribution for all variables except for birthweight on Group 1, birthweight centile on Group 3, pH on Group 2, cortisol on Group 2 and 3, and reelin on Group 1. When studying the differences amongst the three groups of growth restriction severity, a non-parametric test independence of medians was used, either the Kruskal–Wallis test or Mann–Whitney U test depending on the number of groups compared. Additionally, a linear regression was performed in order to adjust for possible confounding variables.

## 3. Results

In total, we obtained 34 subjects, 17 FGR fetuses and 17 AGA fetuses. Of the 17 AGA fetuses, 12 were full-term (37 weeks or more), and of the 17 FGR fetuses, 9 were in Group 2, and 8 were in Group 3. Maternal and neonatal characteristics and differences among the three groups are shown in Table 1.

**Figure 2:**
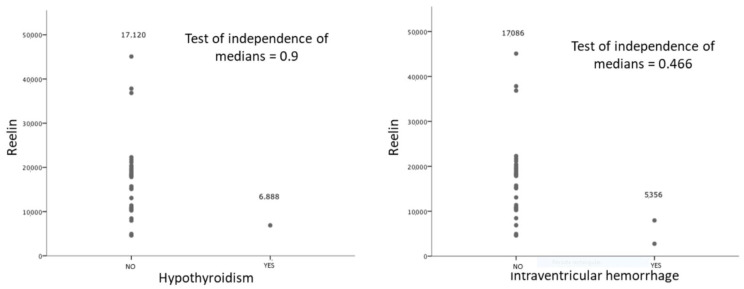
Correlations between reelin levels and intraventricular hemorrhage.

In the absence of any study in the medical literature evaluating reelin in cord blood, the maternal and neonatal variables collected were submitted to univariate analysis. Table 2 shows the results, which indicate that reelin levels were not statistically significantly influenced by the analyzed variables; however, they were clinically lower in the two NBs with ventricular bleeding, although a test of independence of medians showed no statistical significance. (Figure 2).

**Figure 3:**
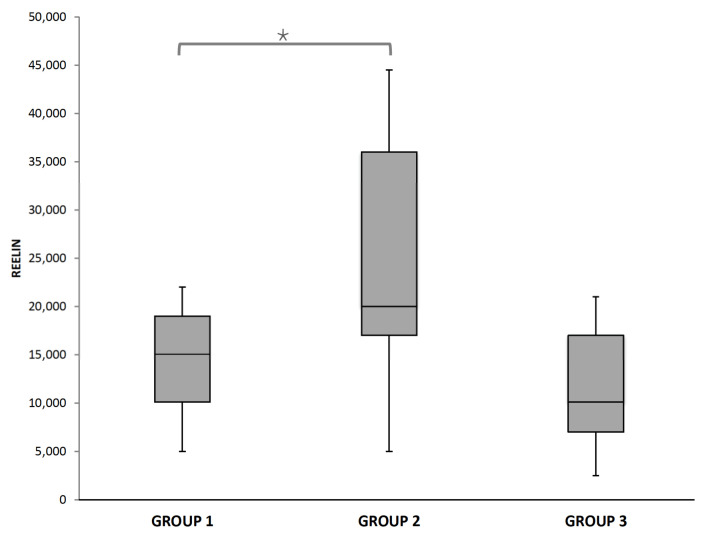
Reelin levels in the three study groups. *Mann–Whitney U test *p* = 0.041, adjusted by gestational age and fetal gender *p* = 0.020.

Reelin levels were higher in growth-restricted NBs affected by cerebral vasodilation (Group 2) than in the other groups, as shown on Figure 3. When comparing medians, differences between the three groups did not reach a statistical significance (Kruskal–Wallis test *p* = 0.083). Exploring further the differences between two groups, we compared medians. The difference reached statistical significance between Group 1 (normal growth) and Group 2 (FGR with cerebral vasodilation), Mann–Whitney U test *p* = 0.041. Nonetheless, when comparing medians from Group 1 and 3, and Group 2 and 3, no statistical difference was revealed, probably due to the small number of cases in Group 3.

Delving into the difference between groups and with the intention of assessing other variables that could have influence over reelin, an adjustment was made with a linear regression for gestational age and fetal sex, obtaining differences between Group 1 and 2, with a higher significance (*p* = 0.020).

**Figure 4:**
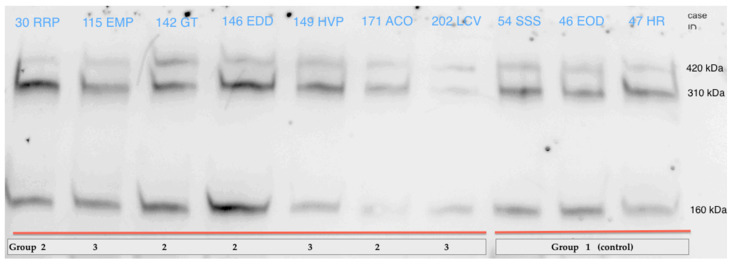
Western Blot of reelin, representative cases, and controls.

A representative sample of the WB analysis is shown on Figure 4.

## 4. Discussion

This is the first study in which peripheral reelin levels in NBs were analyzed and related to the state of pathological intrauterine growth and vascular adaptation. Given the reality of fetuses affected by FGR and impaired neurodevelopment, it was proposed that the concentration of reelin, a protein involved in migration, cortical organization, and synapses during prenatal development of the brain, could vary depending on the type of fetal growth impairment and its adaptive phase.

The descriptive analysis of the sample did not yield any variable that influenced reelin levels. However, the literature describes a signaling mechanism called Reelin-Dab that is influenced by a thyroid hormone. Hypothyroidism reduces reelin expression during neurodevelopment in early stages of noxa [29]. The effect of the administration of exogenous thyroid hormones on reelin levels in rodents has been studied [30]. In our series, hypothyroidism was analyzed separately from the reelin variable due to published evidence, but there was only one case of hypothyroidism in the analyzed sample, and it was being treated. The reelin level in this case was almost three times lower than that of the rest of the cases (6888 versus 17,120), but due to the treatment and the lack of maternal thyroid hormone levels data, no observations could be stated.

Prenatally, other modifying factors of reelin expression have been identified. All related studies have focused on tissue analysis (Northern blot, Western blot, and immunohistochemistry) and rodent models. Alcohol has been shown to induce the expansion of the main neurons that produce reelin (Cajal-Retzius) in Layer I neutrophils [31], and infections caused artificially by lipopolysaccharides induce a reduction in the expression of reelin and a re-organization of pyramidal cells in the hippocampus [32,33]. In our studies, we excluded patients who had taken any type of drug during the initial interview; therefore, this variable was not analyzed. Additionally, the presence of chorioamnionitis was an exclusion criterion.

The variation in reelin in cord blood was studied in a single article evaluating the reelin levels of NBs of mothers treated with serotonin reuptake inhibitors (SRIs). The children of treated mothers had lower cord blood reelin at birth compared to the children of untreated mothers. The differences were more marked in female NBs [34]. This study encourages continued research on cord blood since the differences observed after psychiatric treatment suggest that peripheral reelin levels are a reflection of central nervous system (CNS) levels.

However, it is known that reelin is also produced outside the CNS, such as in the liver, synovial fluid, and platelets, and has other functions [35]; nevertheless, it is during the prenatal stage that its production and role are fundamental in the brain. Immunohistochemistry and electron microscopy showing the presence of reelin in the cerebral endothelium support the passage of this molecule through the blood-brain barrier [36]. Above all, it is well known that the blood-brain barrier is damaged in fetal hypoxic-ischemic injuries [37].

Regarding the field of psychiatric research, reelin levels have been studied in the brains of patients with autism spectrum disorder, bipolar disorder, schizophrenia, Alzheimer’s disease, and major depression, with diverse results since the tissue analyses targeted reelin levels in different regions, such as the hippocampus, frontal cortex, etc. [27]. Most studies report reduced reelin levels in major depression, autism, and bipolar disorder (at the tissue and plasma levels), but not schizophrenia, which is associated with an increase in plasma reelin levels at baseline and during the first episode [38,39], or in the brain tissue of patients with Alzheimer’s disease [40].

Another antenatal factor that has been studied to examine the influence of reelin during prenatal neurodevelopment is hypoxia, due to the theory that fetal stress influences brain development at the cellular, molecular, and epigenetic levels [41]. In rodent postnatal hypoxia models, it has been shown that the expression of reelin in cortical interneurons is increased, especially in the motor and somatosensory cortex [42]. Another postnatal study using a model of ischemic brain injury induced by thermocoagulation demonstrated an increase in the local expression of reelin. That study concluded that reelin played a regenerative role by inducing migration and remyelination starting at neurogenesis in the adult rostral migratory current [43]. In contrast, another prenatal study of a different rodent model of growth retardation caused by protein restriction observed that the expression of reelin by the interneurons of the dentate gyrus was not altered, although other brain areas were not evaluated [44]. Similarly, after postnatal cerebral infarction in rodents, no protective role of reelin was observed during recovery [45]. These differences in results may be because the effect of hypoxia on reelin depends on the CNS area analyzed, the chronicity of the noxa, and the subsequent period analyzed. Golan et al. evaluated the effect of hypoxia in their rodent model, observing that reelin was overexpressed in the cortex and that its concentration was decreased in the hippocampus [15,20]. Another related study by Curristin et al. examined a rodent model subjected to sublethal postnatal hypoxia and showed inhibition of the reelin coding gene in the cortex and hippocampus, with a resulting decrease in reelin in the immunohistochemical analysis [19].

This series of studies leads us to deduce that the adverse perinatal environment causes substantial changes in the expression of reelin in different hypoxia models in rodents.

The variable results may have occurred because reelin can be modified in several ways depending on the severity of the prenatal injury, the CNS area analyzed, and the type of analysis performed (tissue or plasma), and because it is difficult to measure due to its large size, the lack of monoclonal antibodies covering a broad range of epitopes, the presence of multiple products resulting from the proteolysis of the protein, and the inability to test it at its full size once it is secreted and isolated [18].

In our study, the variable analyzed in association with chronic hypoxia was FGR. We did not obtain significant differences between NBs with adequate weights and those with FGR. Arterial pH at birth, which is a reflection of acute intrapartum hypoxia, was also not identified as an influencing variable, but no cases of acidosis in cord blood were identified. When intraventricular bleeding was considered as a marker of severe hypoxia, we did obtain differences despite having only two cases in our sample. The two NBs with intraventricular hemorrhage (IVH) had reelin levels three times lower than the rest of the NBs analyzed (Figure 2). This has to be interpreted with caution as, although the hemorrhage was mild, confined to the germinal matrix, NBs were 25 and 33 weeks old. It may have also influenced the reelin level on Group 3 as all IVH belonged to this group.

To evaluate the role of hypoxia in NBs with FGR further, the degree of Doppler involvement was compared. FGR fetuses possess mechanisms for adapting to the adverse environment of placental insufficiency. These adaptive mechanisms have been observed to occur chronologically, producing cerebral vasodilation first, followed by an increase in pulsatility on the umbilical artery [46]. We observed that the levels of reelin in umbilical venous blood were significantly higher in growth-restricted NBs affected by cerebral vasodilation (Group 2) than in growth-restricted NBs without cerebral vasodilation (Group 3). A physiopathological justification of our finding is the leaky brain theory, which postulates immature cerebral angiogenesis and the uncontrolled passage of proteins through the blood-brain barrier as causes of the elevated concentration of neurological proteins in the plasma of premature infants. Immunohistochemical techniques and active protein transport studies have demonstrated the existence of tight junctions in the neuroendothelium that regulate the flow through the blood-brain barrier from early gestational ages [47] and are damaged by hypoxic injury, leading to greater permeability [37]

The finding of increased reelin in NBs who have adapted through blood flow centralization is of complex significance and supports different interpretations and is limited for the reason of lack of NBs’ follow up. The first is consistent with published articles that highlight the fact that cerebral vasodilation is another stage of intrauterine growth retardation and that, as studies evaluating the neurodevelopment of these fetuses have shown, cerebral compensation does not extend to all the brain functions observed in neurological development tests [48]. For this reason, cerebral metabolic impairment can be observed in NBs with cerebral blood flow redistribution [49] on spectrometry; changes along the same line were observed in the present study, in which reelin was overexpressed in NBs with cerebral redistribution.

## 5. Conclusions

As the CNS is the main source of prenatal reelin, a fetus affected by blood flow centralization can experience greater passage of reelin into the general bloodstream, with uncertain consequences. It is possible that this passage into the bloodstream decreases the level of intracerebral reelin and therefore deteriorates its function as an organizational signal during radial neuronal migration and dendritic maturation. Subsequent studies of the organization and maturation of the CNS and its signal proteins should include a larger sample to allow the formation of uniform and consistent groups, primarily on gestational age and growth restriction severity. Additionally, the advancement of non-invasive imaging and metabolic study techniques will allow in vivo assessments of NBs with growth impairment during different adaptive phases.

## Figures and Tables

**Table 1:** Descriptive groups.

	GROUP 1 (Control) AGA Fetuses N:17	GROUP 2 FGR Fetuses (Pathological MCA Normal UA) N:9	GROUP 3 FGR Fetuses (Pathological UA) N:8	*p*
**Maternal Age (years) m(SD)**	31.35 (4,5)	31.38 (5.7)	30.75 (5.6)	0.91
**Gestational Age (weeks) m(SD)**	38.39 (2.2)	36.04 (2.4)	32.38 (1.8)	<0.001
**Fetal sex (female) *n*(%)**	7 (41)	5 (56)	2 (25)	0.433
**Type of delivery (vaginal) *n*(%)**	10 (59)	2 (22)	0	0.003
**Birth weight (gr) M(IQR)**	3316 (350)	2031 (698)	1446 (620)	<0.001
**Percentile at birth M(IQR)**	63 (38)	2 (5)	2 (1)	<0.001
**pH UA (mean) M(IQR)**	7.26 (0.17)	7.26 (0.13)	7.27 (0.13)	0.530
**Uterine artery PI > p95 *n*(%)**	-	3 (33)	6 (67)	<0.001
**Rupture of membranes > than 12 h. *n*(%)**	4 (24)	0	0	0.133
**Admission to the NICU *n*(%)**	1 (12)	3 (33)	6 (75)	0.001
**Type 1 intraventricular hemorrhage *n*(%)**	0	0	2 (25)	0.005
**Head circumference (cm) m(SD)**	34.4 (1)	31.5 (3)	28.13 (3)	<0.001
**Leukocytes (*n* º/uL) m(SD)**	14,725 (10,200)	15,212 (7625)	11,426 (2800)	0.308
**Cortisol M(IQR)**	8.55 (5)	19.70 (14.7)	1.96 (2.7)	0.035
**Reelin M(IQR)**	15,055 (3954)	20,062(16,645)	10,111(6956)	0.083

m: mean; SD: standard deviation; n: number; gr.:grams; M: median; IQR: interquartile range; UA: umbilical artery; PI: pulsatility index; NICU: neonatal intensive care unit.

**Table 2:** Correlations between reelin levels and clinical study variables.

	Total Reelin M (SD)	*p*
**Maternal Obtetric Characteristics**		
**Age (years)**	**−251 (−928 426)**	0.456
**First pregnancy** No (11)		0.189
	13,318 (6459)	
Yes (23)	17,868(10,273)	
**Disease during pregnancy** (preeclampsia, diabetes, Hypertensión, hypothiroidism) No(26)		0.254

	17,394 (9308)	
Yes (8)	10,968 (6167)	
**Rupture of membranes > than 12 h.** No (28)		0.58
	17,102 (10,043)	
Yes (4)	14,147 (6311)	
**SO4Mg treatment** No (29)		0.94
	16,147(8946)	
Yes (3)	16,619(17,911)	
**Type of delivery** Vaginal (19)	17,288 (9077)	0.43
C-Section (15)	14,532 (10,117)	
**Neonatal Characteristics**		
**Gestational age** (34)	0.18	0.309
**Gender** Male (20)		0.388
	15,111(7554)	
Female (14)	17,919(11,491)	
**Birthweight (g)** (34)	-0.02	0.911
**Lung maturation** Yes (8)		0.728
	15,645	
**Arterial cord pH** (34)	0.111	0.531
**Neonatal reanimation** (32) 0 (17)		0.661
	16,846(10,625)	
I (8)	16,606(6002)	
II (3)	9592(7782)	
III (5)	16,639(11,965)	
**NICU** No (24)		0.592
	16,776(9261)	
Yes (10)	14,880(9661)	
**Intraventricular Hemorrhage** No (32)		0.088
	16,894(9116)	
Yes (2)	5356(3675)	

## Data Availability

The data used to support the findings of the present study are available from the corresponding author upon request.
